# Effectiveness of Vestibular Rehabilitation for Oculomotor Symptoms and Disorientation in Individuals With Cybersickness: A Randomized Controlled Trial

**DOI:** 10.7759/cureus.117964

**Published:** 2026-10-15

**Authors:** Venu D Chafekar, Ajeet Saharan, Dipak S Patade

**Affiliations:** 1 Department of Neuro-Physiotherapy, Sir J. P. College of Physiotherapy, Palghar, IND; 2 Department of Neuro-Physiotherapy, NIMS College of Physiotherapy &amp; Occupational Therapy, NIMS University, Jaipur, IND; 3 Department of Medicine, Adhikari Lifeline Hospital, Boisar, IND

**Keywords:** cybersickness, disorientation, motion sickness, oculomotor, simulator sickness questionnaire, vestibular rehabilitation, virtual reality

## Abstract

Background

Cybersickness is characterized by oculomotor symptoms and disorientation during prolonged screen or virtual reality exposure. The effectiveness of vestibular rehabilitation for these symptoms remains unclear.

Methods

This two-group randomized controlled trial included 50 adults aged 30-50 years with self-reported cybersickness and more than 12 hours of daily screen exposure. Participants were randomly assigned to a vestibular rehabilitation group (n = 25) or a control group (n = 25). The intervention group performed a structured Cawthorne-Cooksey-based vestibular rehabilitation program for 20 minutes per day, six days per week, for six weeks. The control group received ergonomic and screen-hygiene advice. Oculomotor symptoms and disorientation were assessed using the Simulator Sickness Questionnaire (SSQ) at baseline and six weeks.

Results

All 50 participants completed the study. Baseline SSQ scores were comparable between groups. At six weeks, the vestibular rehabilitation group demonstrated greater reductions in oculomotor symptoms than the control group (11.3 ± 3.7 vs. 4.2 ± 3.3; between-group difference, 7.1 points; 95% CI, 5.3-8.9; p < 0.001). Similarly, disorientation scores decreased more in the vestibular rehabilitation group than in the control group (14.3 ± 5.0 vs. 5.3 ± 4.1; between-group difference, 9.0 points; 95% CI, 6.6-11.4; p < 0.001).

Conclusions

A six-week structured vestibular rehabilitation program produced greater reductions in oculomotor symptoms and disorientation associated with cybersickness than ergonomic and screen-hygiene advice alone. These findings support further investigation of vestibular rehabilitation as a nonpharmacological approach for cybersickness.

## Introduction

Cybersickness refers to motion sickness caused by visual input that does not correspond to physical movement; symptoms include headache, disorientation, vertigo, oculomotor discomfort, eye strain, difficulty focusing, and postural instability [1]. These symptoms can occur during exposure to computer screens, mobile devices, or virtual reality (VR) technology. Oculomotor symptoms and disorientation are particularly disruptive and may persist after screen exposure, potentially affecting work performance and sleep [1]. As screen use increases across occupational and nonoccupational settings beyond VR applications, cybersickness is becoming an increasingly relevant occupational health concern [2].

The most widely accepted explanation for cybersickness is sensory conflict theory. According to this theory, a conflict between visual and vestibular or proprioceptive inputs produces a discrepancy between perceived and actual self-motion [3]. This sensory mismatch may contribute to oculomotor discomfort through increased vergence-accommodation conflict and to disorientation through altered sensory integration and vestibular reweighting [4,5].

Vestibular rehabilitation is an exercise-based intervention involving repeated and progressive head and eye movements to promote central compensation [6,7]. It is supported by clinical practice guidelines for peripheral vestibular hypofunction (PVH) and has demonstrated benefits in individuals with dizziness and balance impairments [8,9]. However, traditional vestibular rehabilitation programs have not been adequately studied in individuals with cybersickness, which is primarily considered a visually induced sensory mismatch condition rather than a disorder of the peripheral vestibular system. Therefore, there is a need to directly investigate the effectiveness of vestibular rehabilitation for oculomotor symptoms and disorientation associated with cybersickness.

Kennedy et al. (1993) developed the Simulator Sickness Questionnaire (SSQ), a widely used instrument for assessing symptoms associated with simulator and screen exposure [10]. The questionnaire includes three principal symptom domains: nausea, oculomotor symptoms, and disorientation, in addition to a total severity score. In the present study, oculomotor symptoms and disorientation were prespecified as co-primary outcomes because they represent prominent and functionally relevant components of cybersickness.

This randomized controlled trial (RCT) assessed the effects of a six-week structured vestibular rehabilitation program compared with standard ergonomic and screen-hygiene advice on oculomotor symptoms and disorientation in adults experiencing cybersickness.

## Materials and methods

Study objective and design

The objective of this study was to assess the effectiveness of a structured vestibular rehabilitation program for oculomotor symptoms and disorientation associated with cybersickness. This was a two-arm, parallel-group RCT with blinded outcome assessment. Participants were randomly assigned in a 1:1 ratio to either the vestibular rehabilitation group or the control group. The study was reported in accordance with the Consolidated Standards of Reporting Trials (CONSORT) 2010 statement [11].

Study setting and duration

The study was conducted among participants recruited from Sir J. P. College of Physiotherapy and affiliated corporate workplaces in Palghar District, Maharashtra, India. The study included participant screening and recruitment, baseline assessment, randomization, a six-week intervention period, and final outcome assessment at week 6. The overall study period was eight weeks, including recruitment, baseline evaluation, intervention delivery, and final data collection.

Study timeline

The study was conducted from January 15, 2026, to February 26, 2026. Participant recruitment and screening were performed during the initial phase, followed by baseline assessment and randomization. The intervention was administered for six weeks, and the final outcome assessment was performed at the end of the intervention period.

Eligibility criteria

Participants were eligible if they were adults aged 30-50 years, of either sex, with regular exposure to computer, mobile, or VR screens, daily screen use exceeding 12 hours, and self-reported symptoms of cybersickness confirmed using the SSQ at baseline.

Participants were excluded if they had a history of vertigo or a diagnosed vestibular disorder, cervical spondylosis or cervicogenic dizziness, active motion sickness on the day of assessment, previous ear surgery, current use of ototoxic or centrally sedating medications, or uncorrected visual impairment that could interfere with the assessment of oculomotor symptoms.

Sample size calculation

The sample size was calculated based on an expected effect size of 0.9 derived from previous research evaluating vestibular rehabilitation and dizziness-related outcomes [12]. Assuming 80% statistical power and a two-sided significance level of 0.05, the estimated minimum sample size was 21 participants per group. Allowing for a potential dropout rate of 15%, the required sample size was increased to 25 participants per group, resulting in a total planned sample size of 50 participants.

Randomization, allocation concealment, and blinding

After eligibility was confirmed and baseline assessments were completed, participants were randomly assigned in a 1:1 ratio to either the vestibular rehabilitation group or the control group. The random allocation sequence was generated using a computer-based randomization procedure by an independent researcher who was not involved in participant recruitment, intervention delivery, or outcome assessment.

Allocation concealment was maintained using sequentially numbered, opaque, sealed envelopes that were prepared before participant enrollment. The envelopes were opened after completion of the baseline assessment and confirmation of eligibility.

Blinding of participants and treating physiotherapists was not possible because the intervention involved active therapeutic exercises. However, the outcome assessor was blinded to group allocation and was not involved in intervention delivery. Participants were instructed not to disclose their group assignment to the outcome assessor during follow-up assessments.

Participant flow

A total of 64 participants were screened for eligibility. Of these, nine (14.1%) were excluded because they did not meet the eligibility criteria, and five (7.8%) declined to participate. The remaining 50 participants (78.1%) were randomized in a 1:1 ratio, with 25 participants (50.0%) allocated to the vestibular rehabilitation group and 25 (50.0%) allocated to the control group.

All 50 randomized participants completed the six-week intervention and the final outcome assessment. No participants were lost to follow-up, withdrew from the study, or had missing outcome data. Therefore, all 50 randomized participants were included in the primary analysis.

Interventions

Vestibular Rehabilitation Group

Participants assigned to the vestibular rehabilitation group (Group A; n = 25) received a structured vestibular rehabilitation program adapted from the Cawthorne-Cooksey exercises [13]. The intervention was performed for 20 minutes per day, six days per week, for six weeks. The program included head and eye movements, postural and balance exercises, walking exercises, and gaze stabilization exercises.

Head and eye exercises: Participants performed the exercises in a seated position and completed 20 repetitions of shoulder shrugging, 20 repetitions of right and left shoulder rotations, and 20 repetitions of coordinated head, shoulder, and trunk rotations. Repeated forward-bending exercises were performed while maintaining visual fixation on a designated target. Participants also reached toward the floor and returned to an upright sitting position while directing their gaze toward the floor and then forward. Eye-movement exercises were performed with the head stationary and included horizontal and vertical eye movements, focusing on the nose, and changing the direction of gaze.

Standing and balance exercises: Exercises included sit-to-stand transitions, heel-to-toe standing, tandem standing, standing on a compliant surface, throwing and catching a ball while standing, heel standing, and single-leg standing.

Walking exercises: Participants performed walking in a straight line and walking while slowly rotating the head from side to side.

Gaze stabilization exercises: Vestibulo-ocular reflex (VOR) ×1 exercises were performed by maintaining visual fixation on a stationary target while moving the head horizontally and vertically. Head movement speed was gradually increased from slow to faster speeds according to individual tolerance while maintaining fixation on the target. Exercise difficulty was progressed according to participants’ symptoms, balance performance, and ability to perform the exercises safely.

During the first week, intervention sessions were directly supervised by a physiotherapist. Thereafter, participants performed the exercises independently, with weekly in-person follow-up sessions to assess adherence, monitor symptoms, reinforce appropriate exercise technique, and modify exercise difficulty when required.

Control Group

Participants assigned to the control group (Group B; n = 25) received standard ergonomic and screen-hygiene advice. This included the 20-20-20 rule, optimization of screen brightness and contrast, appropriate screen viewing distance, and scheduled breaks during prolonged screen use. Participants did not receive structured vestibular rehabilitation during the six-week study period and underwent reassessment at week 6.

Outcome measures

The primary outcomes were cybersickness-related oculomotor symptoms and disorientation, measured using the SSQ [10]. The SSQ was completed by a blinded outcome assessor at baseline and at the six-week follow-up.

The SSQ has 16 symptom items rated on a four-point scale from 0 (none) to 3 (severe). The questionnaire includes three symptom domains: nausea, oculomotor symptoms, and disorientation. For the present study, the Oculomotor (OM) and Disorientation (DO) subscale scores were prespecified as co-primary outcomes.

The scores for the OM subscale items were summed, and the total was multiplied by 7.58 to obtain the OM subscale score. The scores for the DO subscale items were summed and multiplied by the respective weighting factor to obtain the DO subscale score. Higher scores indicated more severe symptoms. The treatment effect was evaluated based on changes in the OM and DO scores from baseline to week 6.

During weekly follow-up assessments, any increase in dizziness, disorientation, visual discomfort, or imbalance, as well as any new symptoms occurring during the intervention, was documented. Participants were encouraged to discontinue a task when symptoms became intolerable or they felt unsafe. The supervising physiotherapist evaluated whether the relevant exercise needed to be modified or discontinued.

Statistical analysis

IBM SPSS Statistics for Windows, version 26.0 (released 2018; IBM Corp., Armonk, NY, USA), was used for statistical analysis. Means and SDs were used for continuous variables, while frequencies and percentages were used for categorical variables.

Independent-samples t-tests or chi-square tests were used, as appropriate, to compare baseline continuous and categorical variables between groups, respectively. Within each group, changes in SSQ Oculomotor and Disorientation scores from baseline to week 6 were assessed using paired-samples t-tests. Independent-samples t-tests were used to determine between-group differences in change scores.

Treatment effects were reported as mean between-group differences in change and presented with 95% CIs. All statistical tests were two-sided, and a p-value less than 0.05 was considered statistically significant.

As no randomized participants were lost to follow-up and no outcome data were missing, all 50 randomized participants were included in the primary analysis. No data were missing, and no imputation was required.

Ethical considerations

The study was conducted in accordance with the ethical principles of the Declaration of Helsinki. The relevant institutional ethics committee(s) granted ethical approval before participant recruitment. All participants were informed of the study objectives, procedures, anticipated benefits, and potential risks associated with the intervention. Informed consent was obtained from all participants before enrollment. Participants were informed that they could withdraw from the study at any time without providing a reason or experiencing any adverse consequences. Data collection, analysis, and reporting were conducted in a manner that ensured participant anonymity.

## Results

A total of 64 participants were screened, of whom 50 met the eligibility criteria and were randomized. Twenty-five participants were allocated to the vestibular rehabilitation group and 25 to the control group. All 50 participants completed the six-week intervention and final outcome assessment, with no loss to follow-up or missing outcome data.

The baseline demographic and clinical characteristics of the two groups are presented in Table 1. The groups were comparable with respect to age, sex, daily screen time, and baseline SSQ Oculomotor and Disorientation scores.

**Table 1:** Baseline demographic and clinical characteristics.

Characteristic	Vestibular rehabilitation (n = 25)	Control (n = 25)
Age (years), mean ± SD	38.6 ± 5.9	39.1 ± 6.2
Sex, n (%)		
Male	12 (48.0)	11 (44.0)
Female	13 (52.0)	14 (56.0)
Daily screen time (hours), mean ± SD	13.8 ± 1.4	13.6 ± 1.3
Baseline SSQ Oculomotor score, mean ± SD	22.1 ± 5.5	21.6 ± 5.8
Baseline SSQ Disorientation score, mean ± SD	26.4 ± 7.1	25.9 ± 6.8

The SSQ Oculomotor scores decreased in both groups from baseline to week 6. In the vestibular rehabilitation group, the mean score decreased from 22.1 ± 5.5 at baseline to 10.8 ± 3.9 at week 6, representing a mean reduction of 11.3 ± 3.7 points (p < 0.001). In the control group, the mean score decreased from 21.6 ± 5.8 to 17.4 ± 4.6, representing a mean reduction of 4.2 ± 3.3 points (p = 0.004).

The between-group difference in mean reduction was 7.1 points (95% CI, 5.3-8.9), favoring vestibular rehabilitation (t(48) = 7.42, p < 0.001) (Table 2).

**Table 2:** Oculomotor subscale: within- and between-group comparison. Between-group difference in mean reduction: 7.1 points (95% CI, 5.3-8.9; t(48) = 7.42, p < 0.001).

Group	Baseline, mean ± SD	Week 6, mean ± SD	Mean reduction ± SD	Within-group p-value
Vestibular rehabilitation (n = 25)	22.1 ± 5.5	10.8 ± 3.9	11.3 ± 3.7	<0.001
Control (n = 25)	21.6 ± 5.8	17.4 ± 4.6	4.2 ± 3.3	0.004

The SSQ Disorientation scores decreased in both groups from baseline to week 6. In the vestibular rehabilitation group, the mean score decreased from 26.4 ± 7.1 at baseline to 12.1 ± 4.5 at week 6, representing a mean reduction of 14.3 ± 5.0 points (p < 0.001). In the control group, the mean score decreased from 25.9 ± 6.8 to 20.6 ± 5.9, representing a mean reduction of 5.3 ± 4.1 points (p = 0.001) (Table 3).

**Table 3:** Disorientation subscale: within- and between-group comparison. Between-group difference in mean reduction: 9.0 points (95% CI, 6.6-11.4; t(48) = 7.98, p < 0.001).

Group	Baseline, mean ± SD	Week 6, mean ± SD	Mean reduction ± SD	Within-group p-value
Vestibular rehabilitation (n = 25)	26.4 ± 7.1	12.1 ± 4.5	14.3 ± 5.0	<0.001
Control (n = 25)	25.9 ± 6.8	20.6 ± 5.9	5.3 ± 4.1	0.001

Both prespecified co-primary outcomes showed greater improvement in the vestibular rehabilitation group than in the control group. The between-group treatment effects favored vestibular rehabilitation for both oculomotor symptoms and disorientation, with statistically significant differences in mean change from baseline to week 6 (both p < 0.001). No worsening of oculomotor or disorientation symptoms was reported during the study, and no adverse events attributable to the intervention were recorded.

## Discussion

To minimize the symptoms of visually induced motion sickness, Zhang et al. (2026) reported that progressive vestibular habituation was associated with a reduction in visually induced motion sickness [12]. Their data support the possibility that repeated and graded vestibular exposure may decrease symptom provocation. A six-week vestibular rehabilitation program combining head, eye, balance, gait, and vestibulo-ocular exercises was also used in the present study. However, the current intervention was not a short-term habituation program but rather a structured multimodal rehabilitation program, and changes in the Oculomotor and Disorientation domains were specifically evaluated as treatment effects.

Alashram (2024) systematically assessed RCTs analyzing the effects of Cawthorne-Cooksey exercises on vestibular symptoms and found that vestibular exercise programs had beneficial effects; however, these programs were not always superior to other vestibular exercise programs [13]. The intervention in the present study was adapted from the Cawthorne-Cooksey approach and included additional gaze stabilization, balance, and gait exercises, as well as progressive head-movement exercises. The present study findings should therefore be viewed as the result of the entire structured multimodal vestibular rehabilitation program and not as the result of a specific exercise. This distinction is particularly significant because the intervention targeted multiple components of visual-vestibular integration.

In their abridged Cochrane review, Hillier and McDonnell (2016) reviewed the effectiveness of vestibular rehabilitation in treating dizziness and functional impairment after PVH in one ear [14]. Their results support the use of vestibular rehabilitation as an exercise therapy to improve functional outcomes and reduce dizziness. Although the population in this review had confirmed PVH, the present study population experienced cybersickness without peripheral vestibular dysfunction. Nevertheless, both approaches involved repeated sensory and motor challenges to facilitate adaptation. The present results demonstrate the potential usefulness of vestibular rehabilitation for symptoms related to oculomotor dysfunction and disorientation associated with cybersickness.

Laessoe et al. (2023) explored sensory mismatch and its effects on cybersickness and emphasized the role of discrepancies between visual and postural and/or vestibular inputs [15]. The present findings are relevant to this framework because the vestibular rehabilitation program repeatedly challenged visual-vestibular integration through gaze stabilization, head-movement, balance, and gait exercises. However, the reductions in Oculomotor and Disorientation symptoms may reflect increased tolerance or adaptation to vision-induced sensory conflict. In contrast to the study by Laessoe et al., which investigated factors associated with susceptibility to cybersickness, the present study assessed the effects of a structured therapeutic intervention on symptom severity.

Keshavarz et al. (2023) used the Visually Induced Motion Sickness Susceptibility Questionnaire to demonstrate that susceptibility to visually induced motion sickness-like symptoms varies among individuals [16]. Their findings are relevant to the present study because individual susceptibility and symptom characteristics could affect cybersickness severity. In the present study, the Oculomotor and Disorientation domains were specifically assessed after vestibular rehabilitation rather than susceptibility being assessed before visual exposure. Standardized symptom assessment enabled changes in symptoms to be quantified during the intervention period.

Brown et al. (2022) suggested that the assumption that the SSQ should have a zero or minimal baseline score might not be valid and highlighted the need to account for pre-existing symptoms when interpreting SSQ measurements [17]. This is relevant to the present study because baseline scores may include symptoms such as headache, fatigue, visual discomfort, or mild disequilibrium. Therefore, baseline assessment was essential for characterizing participants before the intervention and measuring changes over the six-week period. The interpretation of treatment effects thus accounted for baseline values and changes from baseline rather than assuming that all observed symptoms were attributable to the visual exposure associated with the intervention.

Geraghty et al. (2017) studied older adults with chronic dizziness who were offered internet-based vestibular rehabilitation and found that structured vestibular exercises could feasibly be delivered remotely with support [18]. This finding provides context for the structure of the present intervention, which consisted of initial therapist supervision followed by independent exercise and regular follow-up. This approach may facilitate implementation outside a controlled clinical setting. However, the populations and clinical contexts differed: the present study included adults with cybersickness-related oculomotor symptoms and disorientation rather than older adults with chronic dizziness.

Whitney et al. (2009) examined the association between gaze stabilization and gait function in people with vestibular dysfunction [19]. Their findings demonstrate the importance of gaze stabilization for maintaining visual stability during head and body movement. Accordingly, the present intervention included progressive balance and gait exercises involving various head movements, as well as horizontal VOR exercises. Improvements in the Oculomotor domain may therefore be associated with improved visual stability during movement, while improvements in Disorientation may be partly associated with better postural control and sensory integration. However, the participants in the present study were not known to have peripheral vestibular dysfunction, which should be considered when comparing the findings.

Aratani et al. (2020) studied the effects of vestibular rehabilitation on patient-reported outcomes in older adults with vestibular disorders and found that vestibular rehabilitation improved symptom-related functional outcomes [20]. Their findings further support the use of individualized vestibular exercise programs to improve patient-reported vestibular symptoms. The present study extends this therapeutic concept to adults experiencing cybersickness symptoms associated with prolonged screen exposure. Both studies used patient-reported outcomes, but the present investigation specifically focused on Oculomotor and Disorientation symptoms of cybersickness. Differences in age, underlying condition, symptom origin, and intervention context should be considered when comparing the studies.

Limitations

This study has several limitations. First, the sample size was relatively small, and participants were recruited from a single geographical area, which may limit the generalizability of the findings to other occupational, demographic, and geographic populations. Second, blinding of participants and treating physiotherapists was not possible because the intervention involved active therapeutic exercises. This may have introduced performance or expectation bias, although the outcome assessor was blinded to group allocation. Third, the primary outcomes were based on the self-reported SSQ, and no objective measures of oculomotor function, vestibular performance, balance, or postural control were included. Therefore, the mechanisms underlying the observed symptom improvements could not be directly evaluated. Fourth, outcomes were assessed only immediately after the six-week intervention, and the durability of the treatment effects remains unknown. Finally, the control group received ergonomic and screen-hygiene advice without an equivalent level of therapist supervision and contact. Differences in attention, motivation, therapeutic expectancy, or interaction with the physiotherapist may therefore have contributed to the observed between-group differences.

## Conclusions

Six weeks of structured vestibular rehabilitation led to greater improvements in oculomotor symptoms and disorientation than ergonomic and screen-hygiene advice alone in individuals with cybersickness. The results indicate that repeated and graded head, eye, balance, gait, and gaze stabilization exercises may alleviate symptoms associated with visually induced sensory conflict. Vestibular rehabilitation could therefore be a promising nonpharmacological treatment for symptoms associated with cybersickness. Further studies using more objective measures of oculomotor, vestibular, and balance function, as well as larger multicenter RCTs with longer follow-up, are warranted to confirm these findings and elucidate the duration and underlying mechanisms of the observed effects.
